# A Deep Learning Based Intelligent Decision Support System for Automatic Detection of Brain Tumor

**DOI:** 10.1177/11795972241277322

**Published:** 2024-09-04

**Authors:** Zahid Ullah, Mona Jamjoom, Manikandan Thirumalaisamy, Samah H Alajmani, Farrukh Saleem, Akbar Sheikh-Akbari, Usman Ali Khan

**Affiliations:** 1 Information Systems Department, College of Computer and Information Sciences, Imam Mohammad Ibn Saud Islamic University, Riyadh, Saudi Arabia; 2 Department of Computer Sciences, College of Computer and Information Sciences, Princess Nourah Bint Abdulrahman University, Riyadh, Saudi Arabia; 3 Department of CSBS, Rajalakshmi Engineering College, Rajalakshmi Nagar, Thandalam, Chennai; 4 Department of Information Technology, College of Computers and Information Technology, Taif University, Taif, Saudi Arabia; 5 School of Built Environment, Engineering, and Computing, Leeds Beckett University, Leeds, UK; 6 Department of Information Systems, Faculty of Computing and Information Technology, King Abdulaziz University, Jeddah, Saudi Arabia

**Keywords:** Brain tumor, detection, CAD, transfer learning, CNN, images

## Abstract

Brain tumor (BT) is an awful disease and one of the foremost causes of death in human beings. BT develops mainly in 2 stages and varies by volume, form, and structure, and can be cured with special clinical procedures such as chemotherapy, radiotherapy, and surgical mediation. With revolutionary advancements in radiomics and research in medical imaging in the past few years, computer-aided diagnostic systems (CAD), especially deep learning, have played a key role in the automatic detection and diagnosing of various diseases and significantly provided accurate decision support systems for medical clinicians. Thus, convolution neural network (CNN) is a commonly utilized methodology developed for detecting various diseases from medical images because it is capable of extracting distinct features from an image under investigation. In this study, a deep learning approach is utilized to extricate distinct features from brain images in order to detect BT. Hence, CNN from scratch and transfer learning models (VGG-16, VGG-19, and LeNet-5) are developed and tested on brain images to build an intelligent decision support system for detecting BT. Since deep learning models require large volumes of data, data augmentation is used to populate the existing dataset synthetically in order to utilize the best fit detecting models. Hyperparameter tuning was conducted to set the optimum parameters for training the models. The achieved results show that VGG models outperformed others with an accuracy rate of 99.24%, average precision of 99%, average recall of 99%, average specificity of 99%, and average *f*1-score of 99% each. The results of the proposed models compared to the other state-of-the-art models in the literature show better performance of the proposed models in terms of accuracy, sensitivity, specificity, and *f*1-score. Moreover, comparative analysis shows that the proposed models are reliable in that they can be used for detecting BT as well as helping medical practitioners to diagnose BT.

## Introduction

The brain is a substantial part of the central nervous control system (CNS) that controls the rest of the human body organs; therefore, brain abnormalities badly affect routine human health.^1^ Brain tumor (BT) is caused due to the abnormal proliferation of brain cells in that the brain’s control management is lost.^2^ In this disease, mass damage occurs to the brain’s neural network, which ultimately damages the function of the brain.^3^ The stages of BT reported are mainly benign and malignant; benign describes the tumor’s early stage when it has a small size and does not spread, while malignancy is a secondary stage that spreads irregularly to surround the brain cells.^4^ According to a report from the National Brain Tumor Society, around 0.7 million patients in the US were diagnosed with BT, of which around 70% were benign and the remaining 30% were in the malignant stage—of which only 36% of patients survived.^5^ Similarly, the World Health Organization^6^ reported that in 2020, cancer is the world’s second highest cause of death, accounting for about 10 million fatalities.^7^ discussed that BT affects 5 to 10 persons per 100 000 of the population in India, and that number is progressing. Over one hundred and twenty distinctive BTs have been identified so far, in which meningioma, glioma, and pituitary are the most common examples,^4^ and their rates for all BTs are 15%, 45%, and 15%, respectively.^8^ The meningioma tumors are potentially obvious among other types of BTs that disturb the brain and spinal cord,^9^ while glioma develops on glia cells and the spinal cord, and pituitary tumors develop on pituitary glands.^10^ BT can be treated using radiotherapy, chemotherapy, or surgical intervention.^11^

With the prompt evolution of radiomics and exploration in image-processing methods over the last decade, various approaches of image elucidation, especially deep learning techniques, have made significant contributions to the medical field.^12,13^ Computer automatic diagnostic systems (CAD) for detecting BT, machine learning,^14^ and deep learning are mostly utilized methods^15,16^ because deep learning, particularly convolution neural network (CNN) models, have achieved more popularity.^17^ These models are normally utilized for classifying medical images and analysis because CNN can extract relevant features from images for precise classification.^8^ CNN based deep learning is a dynamic form of machine learning that significantly establishes indeterminate correlation without implicating much nodal architecture; thus it is a state-of-the-art practice utilized in various fields such as networking, bioinformatics, big data, and medical imaging.^18-20^ In the medical imaging area, significant contributions to artificial intelligence and image processing have been made by CNN,^21^ which has the capability of extracting features from the input image without eradicating spatial information. On the other hand, CNN is more efficient with large datasets, which might be a barrier to finding large data in medical fields; however, transfer learning has addressed the issue with using pre-trained models on large datasets of other domains and helps to achieve promising results on small datasets.^4,22^

This study aims to build an intelligent decision support system for detecting BTs from brain images using deep learning methods. In this regard, 4 deep learning models such as CNN from scratch and 3 transfer learning models such as VGG-16, VGG-19, and LeNet-5 were proposed for detecting BT from medical images. Originally, the dataset was imbalanced; therefore, the data augmentation technique was utilized to balance the data in both classes. Some pre-processing steps were followed to prepare the data prior to training the proposed models. It is worth mentioning that transfer learning was utilized due to the small dataset because in transfer learning, the gained knowledge from models trained on large data helps to achieve high accuracy on small datasets. Moreover, during implementation, a hyperparameter test was performed to select the best-fit parameters in order to achieve the most accurate possible results for detecting BT. The proposed models were evaluated and yielded promising results compared to other state-of-the-art models in the literature. The main contribution is highlighted in the following points:

- Development of a deep learning approach utilizing CNNs for automatic detection of brain tumors (BT) from medical images.
- Implementation and testing of CNN models from scratch as well as transfer learning models (VGG-16, VGG-19, and LeNet-5).
- Utilization of data augmentation to synthetically enhance the dataset for better model training.
- Extensive hyperparameter tuning to optimize the models for the best performance.
- Comparative analysis shows that the proposed models outperform state-of-the-art models in the literature.
- Demonstration of the reliability of the proposed models in aiding medical practitioners in diagnosing BT.

## Related Work

In the literature review for this study, we have explored related work published in different databases. From existing work, it is evident that deep learning models have commonly been used for detection, diagnosis, and analysis of BT. The most commonly used deep learning methods are CNN models. Transfer learning has been a widely utilized methodology for detecting and diagnosing BT because of the non-availability of enough medical images, whereas deep learning models require a large volume of data in order to produce accurate results. Therefore, taking advantage of transfer learning methods is desirable because the models are pre-trained on large datasets and can easily be utilized to gained knowledge with small datasets to produce accurate results. In this section, related work of BT detection from MRI images using deep learning methods has been examined in detail.

An ensemble deep learning model proposed by Alsubai et al^1^ for classifying and detecting BT from MRI image dataset collected from openly available source Kaggle consisted of 253 images. The dataset was pre-processed in that the cropping method was used to remove the unwanted areas from images. Noise elimination was also performed to remove the noise area from images. The ensemble model (CNN-LSTM) was trained using the processed dataset and obtained the maximum accuracy rate of 99.1%. The study of Younis et al^21^ proposed 3 deep learning models (CNN, VGG-16, and ensemble model) to detect BT from MRI images from the same dataset. The dataset was pre-processed and trained in the proposed model. The testing accuracy achieved using CNN, VGG-16, and ensemble models were 89.5%, 97.6%, and 91.29%, respectively. Similarly, the same dataset was used by authors in Febrianto et al^23^ who proposed a CNN model to classify brain MRI images. The authors used the augmentation procedure to enrich the dataset and obtained an accuracy rate of 93%.

A binary classification of BT using CNN models conducted by Alanazi et al^17^ used 3 different datasets, namely datasets I, II, and III. The study proposed CNN from scratch and transfer learning models; initially, dataset I was used for training CNN model for binary classification and achieved 99.33% accuracy. Dataset II was used to categorize types of BT using the transfer learning method and achieved 95.75% accuracy. The performance of the transfer learning model was examined using dataset III, which was unseen data, and tested the model and concluded achieving 96.9% accuracy. Another approach was used by Pereira et al,^24^ who proposed the CNN model for binary classification of BT using whole brain and tumor region of interest (ROI) images of the BRATS dataset. The accuracy rate obtained for the whole brain using whole images and brain mask was 89.50%, while ROI accuracy rate for the whole image was 87.70%, and the brain mask achieved a higher accuracy rate of 92.98%.

A BrainGAN framework was proposed by Alrashedy et al^25^ using generative adversarial network (GAN) architecture and deep learning models to produce and classify brain MRI images. The dataset was collected from Kaggle and contained 400 images. Augmentation of images was performed using vanilla GAN and deep convolution GAN (DCGAN) to generate synthetic images. The processed dataset was utilized for training 3 proposed models: ResNet152V2, MobileNetV2, and CNN. The results concluded that ResNet152V2 outperformed other models with a high accuracy rate of 99.09%. A pretrained GAN model was proposed by Ghassemi et al,^26^ who utilized 2 different datasets of brain MRI images collected from hospitals in China. The first dataset consisted of 3064 images of different types of BT, while the second dataset consisted of 373 images of the whole brain used for dementia study. Augmentation techniques were utilized to generate synthetic images, and normalization was used to scale the images with a range of −1 and 1. The results showed a high accuracy rate of 98.57%. Similarly, a hybrid method of combining machine learning with deep learning was utilized by Senan et al^27^ for detecting BT from MRI images. The dataset consisted of 3060 images distributed into 4 different classes. Several pre-processing steps were taken to enhance the images. AlexNet and ResNet-18 were combined with a support vector machine (SVM). Feature extraction was performed using deep learning methods, and the labeling was accomplished using SVM and softmax. AlexNet combined with SVM bettered other approaches with an accuracy rate of 95.10%. Recently, a study by Amin et al,^28^ an ensemble transfer learning and quantum variational classifier (QVR) model was proposed for detecting BT and was trained using locally gathered images, Kaggle, and BRATS datasets. The achieved detection rate reported in this study reached above 90%.

A study by Shelatkar et al^7^ discussed the use of transfer learning in identifying and classing BT from MRI images using the BRATS dataset and proposed a YOLOv5 deep learning model. During implementation, authors have used different variants of YOLOv5 models, with the highest accuracy rate ranging from 82% to 92%. A study conducted by Alqudah et al^3^ classified brain MRI images into 3 classes of BT using deep learning. The dataset was collected from a freely available source containing 3064 MRI images. The projected CNN model was developed using the image dataset and yielded a highest accuracy rate of 98.93%. Another attempt was made by Bingol and Alatas,^11^ who classified BT through MRI images using the transfer learning method. The dataset was acquired from Kaggle and consisted of images of both patients with BT and healthy people. For detecting BT, 3 transfer learning models were proposed—that is, Alexnet, Googlenet, and Resnet50. Resnet50 outperformed other models and yielded the highest accuracy of 85.71%. Recently, a multi-class classification was performed in Filatov and Gnah^29^ using the transfer learning method, for which the models proposed and used in the study contained ResNet50, EfficientNetB1, EfficientNetB7, and EfficientNetV2B1. The data gathered from Kaggle consisted of 7022 MRI brain images. The dataset was synthetically augmented and trained the proposed models, and the achieved results showed that EfficientNetB1 outperformed other models and reported training and validation accuracy results of 87.67% and 89.55%, respectively. Similarly, the study conducted by Kabir Anaraki et al^30^ classified BT images using deep learning and genetic algorithms (GA), and its datasets were gathered from different available databases. Augmentation to the dataset was performed by synthetically adding images to the training set. The integrated framework of CNN-GA was trained and achieved the highest accuracy of 94.2%.

Similarly, a CNN model was proposed by Badža and Barjaktarović^31^ for classifying BT into 3 types and tested on an MRI dataset, which was augmented synthetically and pre-processed before training the models. The model achieved a high accuracy rate of 96.56% when tested on the augmented dataset using 10-fold cross-validation. Another study by Mehrotra et al^32^ used a transfer learning approach for classifying BT images as benign or malignant. A dataset consisting of 696 MRI images was used to train SqueezeNet, GoogleNet, ResNet-101, AlexNet, and ResNet-50. Among the proposed models, AlexNet outperformed the others with an accuracy rate of 99.04%. A 3D CNN model was proposed^33^ for microscopic BT detection and classification. The best fit features were selected using feature extraction through a transfer learning model. The final classification was performed using a feedforward neural network based on the selected features. The study utilized 3 versions of the BRATS dataset (2015, 2017, 2018). The model was trained using 3 datasets and reported accuracy rates of 98.32%, 96.97%, and 92.67% for the respective mentioned datasets.

A study conducted by Badjie et al,^34^ used BraTS2020 dataset and achieved 99.62% accuracy using AlexNet architecture. A CADD scheme was developed by Rajinikanth et al^35^ to classify BT into Glioma with optimal accuracy. The feature extraction from the TCIA MRI dataset was performed using several approaches and the highest accuracy rate of above 98% was achieved using the SVM-Cubic classifier. A U-Net CNN was applied by Maqsood et al^36^ to recognize the brain images into meningioma and non-meningioma. The proposed model yielded the highest accuracy rate of 98.59%. An approach of Harris Hawks optimized convolution network proposed by Kurdi et al^37^ to recognize the BT. The proposed model was applied to the brain MRI dataset and yielded 98% of accuracy. A multimodal framework was proposed by Khan et al^38^ to classify the BT. The proposed framework was applied to 3 different datasets and achieved 95.14, 94.89, and 95.94% of accuracies.

## Deep Learning Architectures

Deep learning and computer vision have evolved in the healthcare industry in that they use artificial intelligence to apply interpretations to prognostic and decision-making-related problems by utilizing some algorithms that analyze certain measures in digital images and videos. Deep learning is not an individual methodology, but rather consists of several algorithms and topologies applied to a wide range of tasks. In this study, we used some architectures of CNN that have been developed in recent years for predictive analysis and decision-making in the healthcare industry. The following sections discuss these architectures in detail.

### CNN architecture

A CNN is a multilayer NN architecture that was originally developed for handwritten character recognition.^39^ The architecture of CNN was naturally stimulated by the animal visual cortex and is mainly beneficial in image-processing applications.^40^ A CNN is a distinct type of deep learning NN that has performed with exceptional success in image-related tasks and their applications.^41^ The performance of CNNs is heavily dependent on the architectures—for example, number of layers used, combinations of these layers, and specified parameters used in the layers.^42^ The early layers of CNN extract the features, and the later layers integrate these features into a high level of elements in the input.^43^

**Figure 1.**
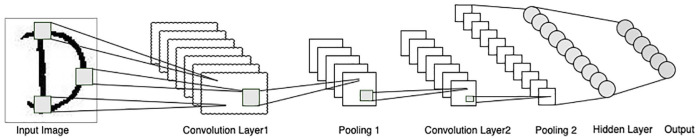
CNN from scratch.

A CNN is comprised of the different layers—that is, input, hidden, and output layers. Figure 1 shows a sample CNN architecture for handwritten characters. The input image is processed in a grid-shaped topology that is represented with the dimensions of (*D* × *D* × *C*), where *D* denotes the number of pixels in an input image, and *C* denoted the numeral of channels per pixel.^44^ The input shape is passing through various layers to generate the output.

#### Convolutional layers

The initial layer in a CNN is a convolutional layer that takes input from the input layer and convolves it with filter size to extract a feature map. The mechanism of creating features is a series of steps performed in a convolutional layer. First, the number of pixels of the same size as the filter size starting from the top left side of an input image is selected to perform a dot product between them and aggregate them, move the filter 1 pixel ahead to the right side, and repeat the process until all the pixels cover the image.^45^ Moreover, many of these filters operate contemporarily on the same input in that every filter predicts distinct parameters.^46^ In each convolutional layer, the features map is then passed through an activation function for nonlinearity, using several activation functions such as ReLu, leaky ReLu, tanh, and sigmoid conferred in the literature; among these, ReLu is a frequently utilized activation function for nonlinearity in convolutional layers.^47^

#### Pooling layers

The layers are typically used after convolutional layers and used to reduce the resolution of an image, referred to as the pooling layer. These layers reduce the dimensionality of an image while preserving the essential features in the features map. This layer is used in special types such as max pooling and average pooling with a specific window size.

#### Fully connected (FC) layers

Fully connected layers in a CNN occur after the pooling layers, which consist of several neurons in which the neuron of 1 layer is connected to each neuron in the next layer. The prediction or classification of images is based on these neurons in the output layer. In fully connected layers, activation function such as ReLu, sigmoid, etc. are used; however, output layer utilized the softmax as an activation function.

### Transfer learning

This is an ML approach in which the knowledge gained from a model developed on a large and generalized dataset in one task can be reutilized as a base to solve the problem of another task. There are several transfer learning architectures, but we will discuss only those used in this study in the following sections.

#### VGG-16

The model architecture of VGG-16 uses 16 layers and belongs to the visual geometry network (VGGnet). The VGGnet is a CNN based model that was used on the ImageNet dataset. A typical VGG-16 is constituted of 5 convolutional blocks and 3 FC layers. The filter size in each convolutional layer was used of size (3 × 3) with the stride of pixel 1 and the same padding, followed by a max pooling of size (2 × 2). In fully connected layers, the first 2 layers consisted of 4096 neurons, and the third layer consisted of 1000 neurons. The ReLu activation function was used in all convolutional and FC layers of VGG-16 except the output layer, which used softmax for image classification.

#### VGG-19

This is another VGGnet deep learning model that is 19 layers deep in the model architecture. Just as with VGG-16, VGG-19 is composed of 5 convolutional blocks and 3 fully connected layers. The filter size of (3 × 3) convolution was used with a stride of 1 pixel in order to cover the whole image. For dimensionality reduction in VGG-19, a max pooling of size (2 × 2) was used to reduce the volume of an image, and spatial padding was used to preserve the spatial resolution of an image. For the computational time improvement and to handle the linearity in the image the ReLu activation function is used. In FC layers, 4096 neurons were used in the first 2 layers while the output layer consisted of 1000 neurons with SoftMax activation for image classification.

#### LeNet-5

The model architecture of LeNet-5 has a total of 5 layers, hence the name Lenet-5. The model was originally developed for handwritten character recognition, as discussed in Lecun et al^48^ This model is composed of 3 convolutional and 2 FC layers. The 3 convolutional layers used 6, 16, and 120 filters, respectively. In each convolutional layer, a filter size of (5 × 5) was used, followed by an average pooling of size (2 × 2). The activation function used in each convolution layer is tanh. The first fully connected layer consisted of 84 neurons that were connected to 10 neurons of the softmax layer for classifying/recognizing the handwritten characters.

## Proposed Models

In this study, we have proposed different neural network models such as CNN from scratch and transfer learning to include VGG-16, VGG-19, and LeNet-5.

### CNN from scratch

**Figure 2.**
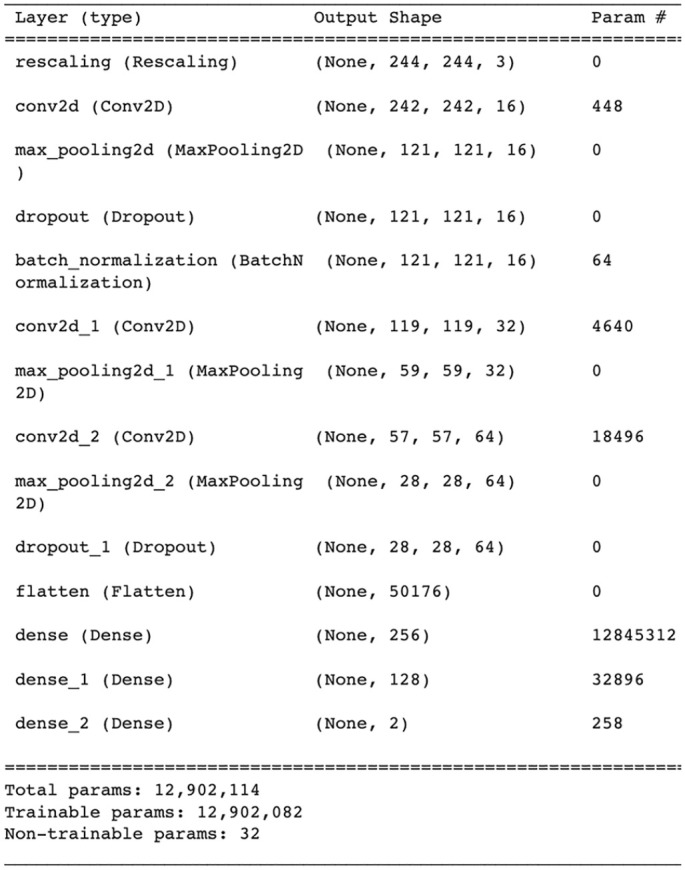
Summary of CNN model from scratch.

The architecture of the proposed CNN from scratch includes convolutional layers, pooling layers, and FC layers. The input image in the convolutional layer was in RGB form of size (244 × 244). Three convolutional layers of 16, 32, and 64 filters of images were used with respect to (3 × 3) kernel size. In each convolution layer, ReLu was used as an activation function and valid padding. After each convolutional layer, max-pooling of stride 2 was used for the dimensionality contraction of the images. Model overfitting was avoided using dropout operations and batch normalization, which were used after the first and third pooling layers. The model was flattened and followed by the fully connected layers, in which 2 layers of size 256 and 128 neurons, respectively, were built and ReLu serving as an activation function. The softmax layer comprised 2 neurons that classified the images according to their classes. Figure 2 summarizes the proposed CNN model from scratch.

### Transfer learning models

**Figure 3.**
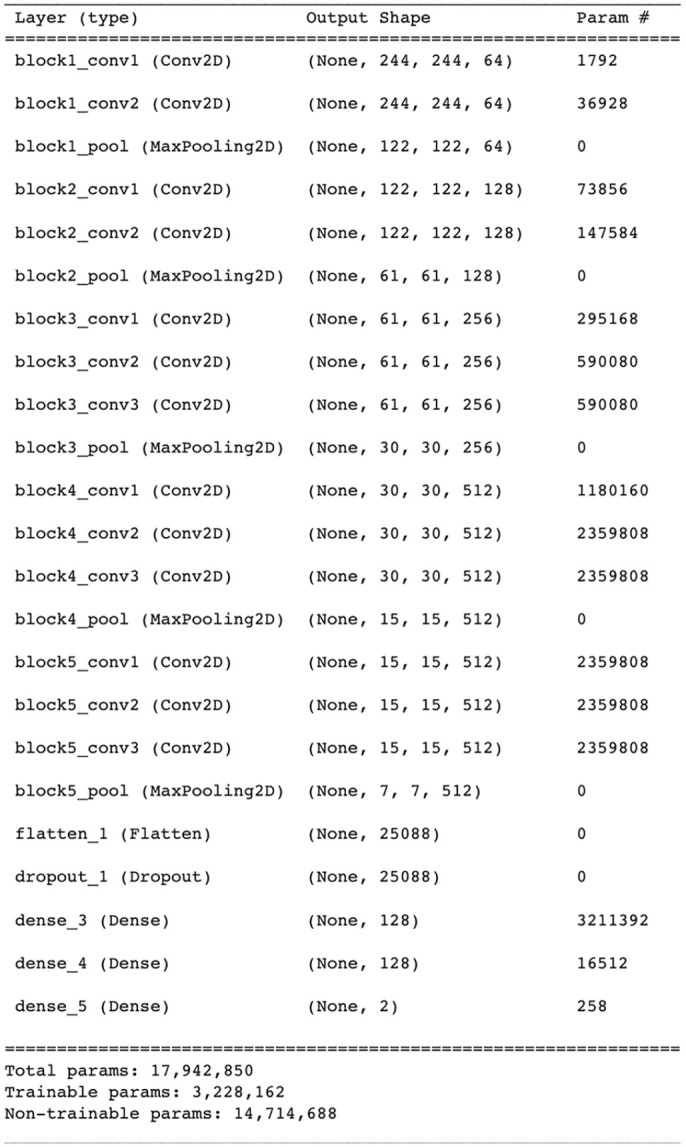
Summary of VGG-16.

In transfer learning, we used 3 different models—that is, VGG-16, VGG-19, and LeNet-5. In VGG-16, we used the standard architecture containing 5 convolutional blocks and 3 fully connected layers (16 layers deep). The filter size of (3 × 3) convolution was used with a stride of 1 pixel to cover the entire image. The dropout operation was performed before the fully connected layers in order to prevent the VGG-16 model from overfitting. For the purpose of this study, the first 2 FC layers were set to 128 neurons each with ReLu function, while the last layer was set to 2 neurons to classify the brain images according to 2 distinctive classes. The summary of VGG-16 is shown in Figure 3.

**Figure 4.**
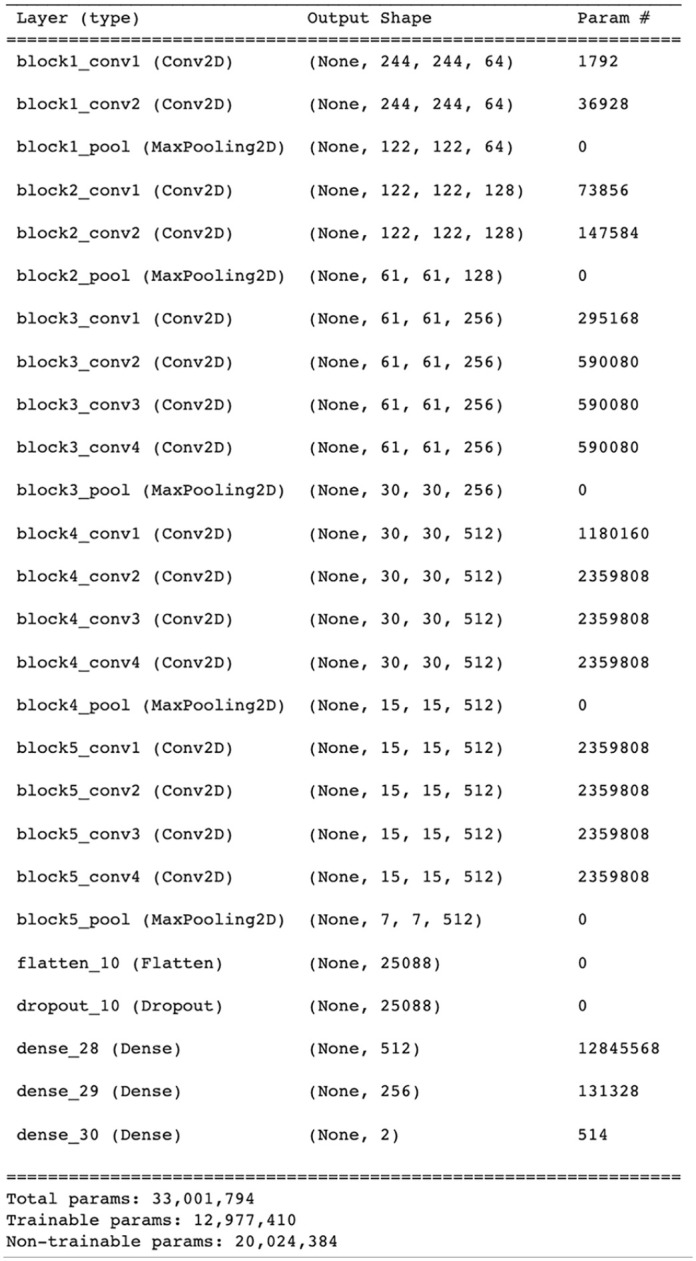
Summary of VGG-19.

Similarly, we used the standard architecture of VGG-19 consisting of 5 convolutional blocks and 3 fully connected layers (19 layers deep). The dropout operation was performed before the fully connected layers to prevent the VGG-19 model from overfitting. In FC layers, the first layer used 512 channels and the second layer used 256 channels, while ReLu activation was used in VGG-19. The softmax layer used 2 channels to sort the images into their respective classes. Figure 4 shows a summary of VGG-19.

**Figure 5.**
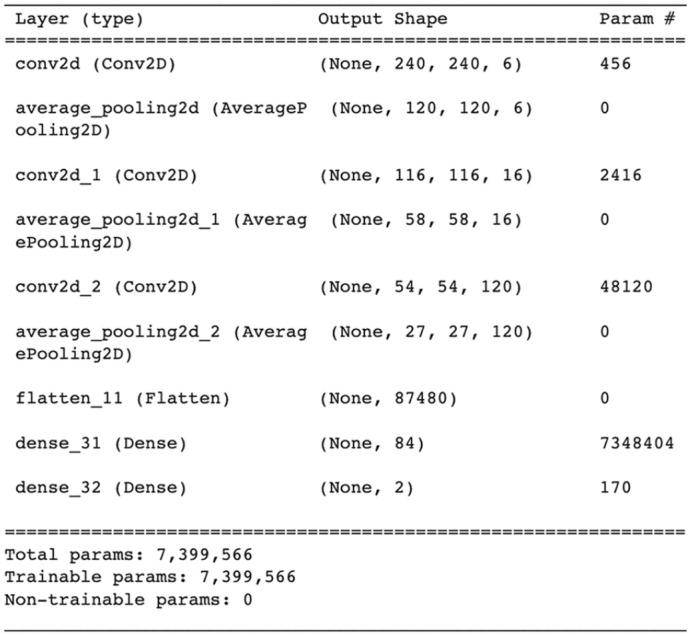
Summary of LeNet-5.

The third model utilized in transfer learning was LeNet-5. In this model, we used 3 convolutional layers of filters (6, 16, and 120, respectively) with kernel sizes of (5 × 5). The activation function used in each convolution layer was tanh. Next to each convolutional layer, an average pooling of size (2 × 2) was used. In FC layers, the first layer consisted of 84 neurons and used tanh as an activation function, while the softmax layer consisted of 2 neurons used for the classification of images in their relevant classes. A summary of LeNet-5 is shown in Figure 5.

## Dataset Description

**Figure 6.**
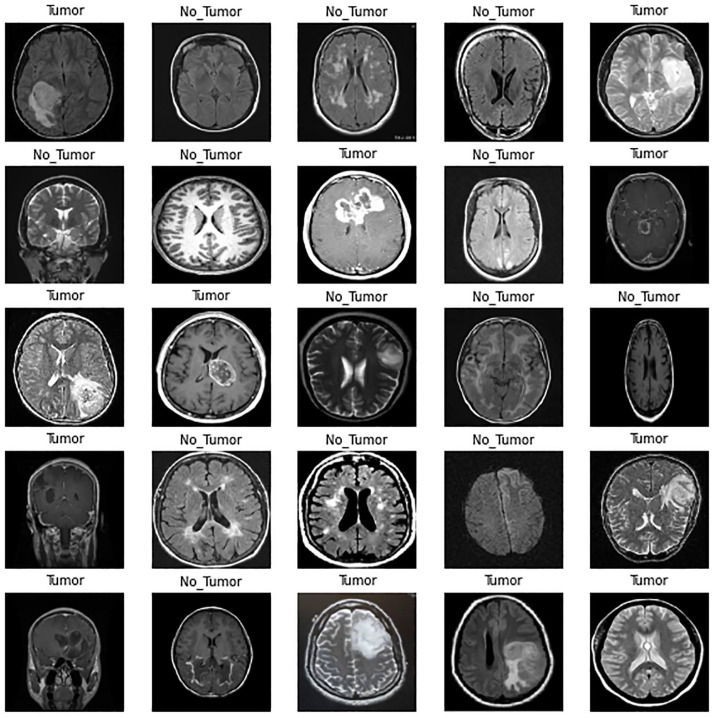
Sample of images in a dataset.

The data used in this work acquired from Kaggle,^49^ which is the openly available website for data scientists and researchers. The dataset entailed of brain images of patients diagnosed with a brain tumor. Originally, the dataset consisted of a total of 4600 images categorized into 2 distinct classes: patients diagnosed with brain cancer and patients with no cancer. The number of patients that are diagnosed with cancer is 2513, while the number of healthy patients is 2087, as shown in Figure 5. Moreover, Figure 6 shows the images present in the dataset.

### Data augmentation

Since a large amount of training data is required for reliable prediction in deep learning models; therefore, data augmentation is an effective technique for increasing data size.^50^ In this approach, the existing data is artificially increased to build a better-generalized model^43^ and prevent overfitting problems in model building.^39^ Similarly, the imbalanced distribution of data in different classes can also affect the classification process in that minority classes make the least impact to the whole accuracy of the model, thus yielding biased results.^51^ The minority classes should be mounted with more data to balance them for fair results.^52^ On the other hand, it can be challenging to find real-world data for particular classes. Therefore, data augmentation is a feasible solution to generate and enrich data artificially in the minority classes.

**Figure 7.**
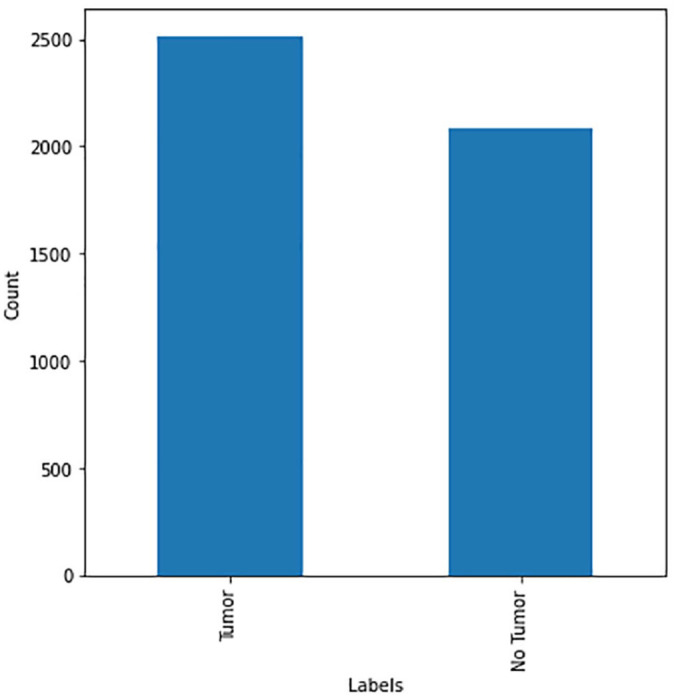
Number of images per class.

**Figure 8.**
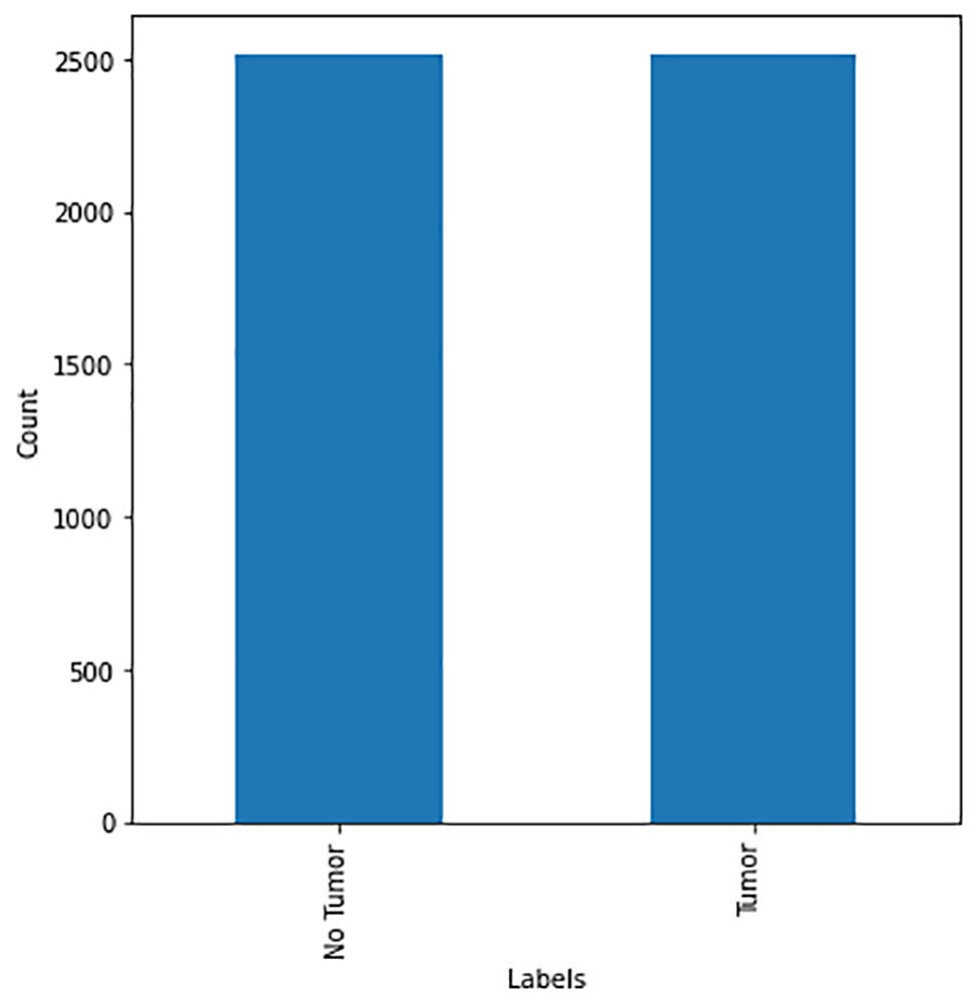
Data distribution after data augmentation.

As shown in Figure 7, the dataset is slightly imbalanced. Therefore, data augmentation was used to balance both classes to achieve accurate results from the prediction models. In this study, several data augmentation methods such as flipping, rotation, and cropping were used in the minority class on randomly selected images to extend the size of the class. Figure 8 shows data distribution after data augmentation.

## Experimental Setup

The proposed architectures were built using the augmented dataset, which was split into three different sets: training, testing, and validation at the proportion of 70%, 20%, and 10%, respectively. The proposed models were optimized using Adam optimizer, and categorical cross entropy was used to measure loss, while the metrics were set to measure the accuracy of the proposed models. The batch size for each model was set at 32, which is an optimal number after several experiments on different numbers. In the proposed models, CNN from scratch was executed with 100 epochs, and the transfer learning models were executed with 50 epochs. The difference in the number of executions was due to saving the execution time for training and testing other models excluded from this study because their results were not promising. The whole parameters that are used in the proposed models were decided after hyperparameter tuning. The tuning of hyperparameter was executed using random search to determine the optimum parameters for model building. Moreover, the proposed models were built using Google colab in a GPU environment. The best classifiers according to the accuracy metrics of the proposed models were saved to the directory for future use. The proposed models were loaded from the directory, and the performance of each model was evaluated using accuracy, precision, sensitivity, *f*-score, and specificity as represented in the following equations.

(1) $$Accuracy=\frac{TP+TN}{TP+TN+FP+FN}$$

(2) $$Precision=\frac{TP}{TP+FP}$$

(3) $$Recall\;or\;Sensitivity=\frac{TP}{TP+FN}$$

(4) $$F-measure=\frac{\left(2*Precision*Recall\right)}{Precision+Recall}$$

## Result and Discussion

**Figure 9.**
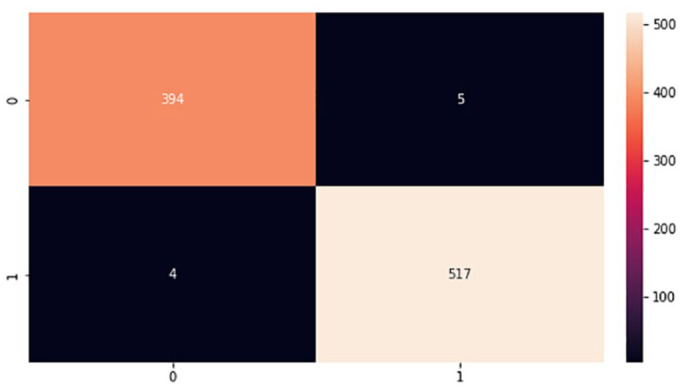
Confusion matrix of CNN from scratch. 0 represents no tumor while 1 represents a tumor.

**Figure 10.**
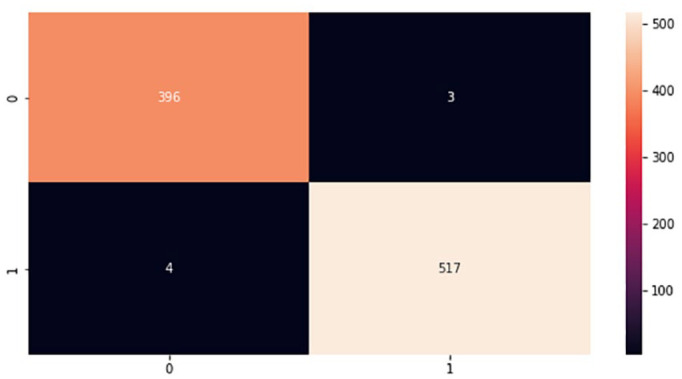
Confusion matrix of VGG-16. 0 represents no tumor while 1 represents a tumor.

**Figure 11.**
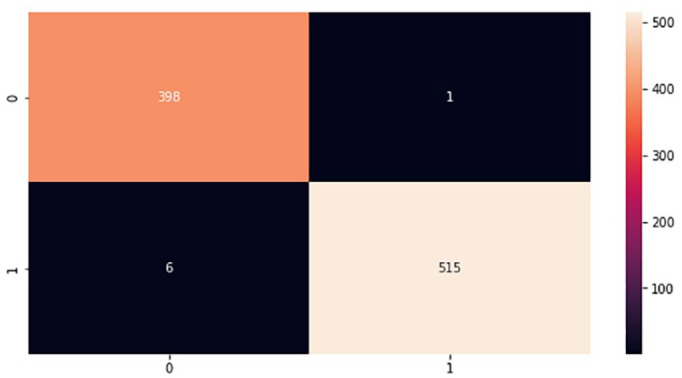
Confusion matrix of VGG-19. 0 represents no tumor while 1 represents a tumor.

**Figure 12.**
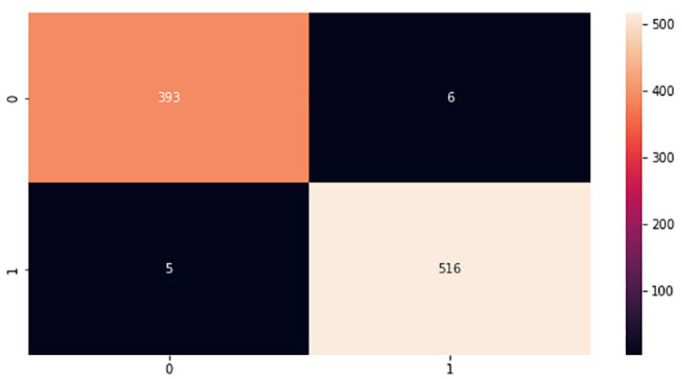
Confusion matrix of LeNet-5. 0 represents no tumor while 1 represents a tumor.

In the implementation phase, the results achieved from the proposed models were finalized based on the best classifiers according to accuracy metrics after repeating the experiments several times testing a different set of parameters. The proposed models were tested using a test set that was 20% of the whole brain images dataset. Moreover, during the experiments, a confusion matrix for each proposed model was computed to provide important values such as true positive (TP), false positive (FP), true negative (TN), and false negative (FN), which are processed based on correctly and incorrectly classified brain images.^53^ These values are the basis for computing model performance such as accuracy, specificity, precision, f-measure, and sensitivity, which is referred to as the classification report of the prediction models. Figures 9 to 12 show the confusion matrixes of the proposed models.

The testing accuracies of the proposed models accomplished according to equation (1) are shown in Table 1. Although, there are FP and FN values in the confusion matrix which leads to type1 and type2 errors, respectively. However, the above confusion matrixes show relatively low number of both type1 and type2 errors, indicating that the model is performing well in accurately identifying brain tumors.

**Table 1.** Accuracies of proposed models.

Proposed model	Accuracy rate (%)	Misclassification rate (%)
VGG-16	99.24	0.76
VGG-19	99.24	0.76
CNN from scratch	99.02	0.98
LeNet-5	98.80	1.20

As in Table 1, the overall performance of the proposed models in terms of accuracy is higher; however, VGG-16 and VGG-19 outperformed the other models. The best fit of VGG-16 and VGG-19 is due to the depth of layers deeper than that of CNN from scratch, which is 6 layers deep, and similarly LeNet-5, which is 5 layers deep. Moreover, the misclassification of VGG-16 and 19 is only 0.76%, showing the model’s reliability to be used for the automatic detection of BT and can also support medical practitioners in diagnosing the disease. Similarly, the proposed models were evaluated using other measures exhibited in Table 2.

**Table 2.** Classification report of proposed models.

Model		Precision	Sensitivity	Specificity	*F*1-score
VGG-16	No_Tumor	0.99	0.99	0.99	0.99
	Tumor	0.99	0.99	0.99	0.99
	Accuracy				0.99
	Macro avg	0.99	0.99	0.99	0.99
	Weighted avg	0.99	0.99	0.99	0.99
VGG-19	No_Tumor	0.99	1.00	0.99	0.99
	Tumor	1.00	0.99	0.99	0.99
	Accuracy				0.99
	Macro avg	0.99	0.99	0.99	0.99
	Weighted avg	0.99	0.99	0.99	0.99
CNN from scratch	No_Tumor	0.99	0.99	0.99	0.99
	Tumor	0.99	0.99	0.99	0.99
	Accuracy				0.99
	Macro avg	0.99	0.99	0.99	0.99
	Weighted avg	0.99	0.99	0.99	0.99
LeNet-5	No_Tumor	0.99	0.98	0.99	0.99
	Tumor	0.99	0.99	0.99	0.99
	Accuracy				0.99
	Macro avg	0.99	0.99	0.99	0.99
	Weighted avg	0.99	0.99	0.99	0.99

The values in Table 2 show the reliability of models in regards of sensitivity, precision, specificity, and f1-score. Overall, the proposed models provide best-fit results for detecting BT patients based on brain scans. However, for comparison of results with the state-of-the-art models in the literature, we will compare the results of either VGG-16 or VGG-19, since both models yield the same results. Table 3 shows the results comparison of the proposed models with the existing models in the literature.

**Table 3.** Comparison of the proposed model.

Reference	Dataset	Model	Accuracy (%)	Precision (%)	Recall (%)	*F*1-score (%)	Specificity (%)
Alsubai et al^1^	Kaggle	CNN-LSTM	99.1	98.8	98.9	99.0	—
Younis et al^21^	Kaggle	CNN	89.5	—	89.5	91.76	—
		VGG-16	97.6	—	94.4	92.6	—
		Ensemble	91.29	—	91.4	91.29	—
Alrashedy et al^25^	Kaggle	ResNet152V2	99.09	99.12	99.08	—	—
Senan et al^27^		AlexNet-SVM	95.10	—	95.25	—	98.50
Filatov and Gnah^29^		EfficientNetB1	89.55	—	—	—	—
Bingol and Alatas^11^	Kaggle	Resnet50	85.71	77.78	82.35	80	87.50
		Alexnet	79.59	77.78	70	73.68	86.21
		Googlenet	83.67	83.33	75	78.95	89.66
Rai and Chatterjee^54^	Kaggle	LeU-Net (cropped images)	98	97	100	98	95
		LeU-Net (uncropped images)	94	97	94	95	95
Rehman et al^33^	BRATS 2015	3D CNN	98.32	—	—	—	—
	BRATS 2017		96.97	—	—	—	—
	BRATS 2015		92.67	—	—	—	—
Mzoughi et al^55^	BRATS	3D deep CNN	96.49	—	—	—	—
Febrianto et al^23^	Kaggle	CNN	93.38	—	—	93.15	—
Kabir Anaraki et al^30^		CNN-GA	94.2	—	—	—	—
Proposed	Kaggle	VGG-16, VGG-19	99.24	99	99	99	99

As shown in comparison Table 3, the proposed models outperformed the state-of-the-art models in the literature. We have compared the proposed with the recent models developed for BT detection; however, for fair comparisons, we include models published since 2019. Moreover, to compare the architecture and methodology used in the proposed model with the existing models, we will discuss some of these models as shown in Table 3.

In Younis et al,^21^ the study used a deep learning approach to detecting BT in which VGG-16 outperformed other proposed models and showed testing accuracy of 97.6%, 94.4% recall, and 92.6% F1-score. The utilized dataset containing 253 MRI images. Various pre-processing steps were followed to systematize images for achieving optimal results, including normalization, elimination of minor patches of deformation, and setting the image outline by cutting the top, bottom left, and right sides. Moreover, the dataset was processed to eliminate the dark margin and enhance the quality of images using different algorithms. The input images were resized to a size of (224 × 224), which is standard for a VGG-16 model. In the model architecture, batch normalization, temporal sub-sampling, and receptive fields were combined.

An ensemble deep learning for detecting BT using MRI images was proposed by Alsubai et al,^1^ in which the same dataset of brain MRI images was utilized for analysis. The images were processed such as normalization, resizing, extreme point calculation, thresholding, cropping, and bicubic interpolation. The proposed model is a hybrid of CNN and LSTM, as CNN was utilized for feature extraction and LSTM was used as a classifier. The overall architecture of the proposed hybrid model was a 16-layer network consisting of 3 convolutional, 7 pooling, 4 convLSTM, 1 fully connected, and 1output layers. In the model architecture, the first block of 3 conv and pooling layers represent CNN, while the next block of 4 convo and pooling layers is used as LSTM. The input image size of (64 × 64) was used in the CNN to create the feature map, while the output of the first block was of size (56 × 56) for the second block, LSTM. The model was trained and evaluated to reach the highest accuracy score of 99.1%, precision of 98.8%, recall of 98.9%, and f-score of 99%.

In Rai and Chatterjee^54^ proposed a novel model referred to as LeU-Net, as it is inspired by the U-net and Le-Net models. The model architecture is split into 3 main parts. The first part contained 2 convo2d and 2 pooling layers. The input image size used was resized to (224 × 224). In the first convo2d layer 32 filters of kernel size (3 × 3) were used, while 64 filters of the same kernel size were used in the second convo2d layer. The second part consisted of 2 convo2d layers next to a transpose layer, which is used to reverse the dimension shrunk by the convo layer. In this part, the transpose layer with 64 filters of size (2 × 2), the first convo2d layer of the same filters with size (3 × 3), and the second convo2d with 32 filters of size (3 × 3) were used. In the third part, 1 FC layer and 1 output layer were used of 128 and 2 neurons, respectively. The activation function used throughout the layers was eLU, except for the output layer. The dataset used in this study was the same as that used in the first 2 studies. Data augmentation was implemented to add to the number of images synthetically and other pre-processing steps like image distribution, resizing of images, and cropping of images. Moreover, the proposed model was assessed on both cropped and uncropped images and reported the results as shown in Table 3.

The study of Mzoughi et al^55^ proposed a 3D CNN model consisting of 8 convolutional and 3 FC layers of BT classification. The model architecture represented the number of filters in 8 convo layers with a kernel size of (3 × 3) 32, 64, 128, 256, 256, 128, 64, and 32, respectively. After convo layers 2, 3, and 4, a pooling layer was used except for the first and fifth convo layers, and batch normalization was utilized throughout the convo layers except layer 8. In convo layers 6 and 7, up-sampling was used while dropout operation was utilized in the fully connected layers. In the FC layers, the first 2 layers with 256 channels each and an output layer with 2 channels were used for the classification of images. The dataset BRATS 2018 was utilized for testing the model. Pre-processing steps such as intensity normalization, resizing, and data augmentation were performed to enhance the dataset quality for training purposes. The model was compiled using a stochastic gradient descent (SGD) optimizer. The reported results are shown in Table 3.

As discussed above, various researchers have proposed novel methods for detecting and identifying BTs from MRI images. The size of datasets and detailed architecture of models in existing studies were also discussed to elucidate their methodologies and proposed models. In the first 3 studies, Alsubai et al,^1^ Younis et al,^21^ and Rai and Chatterjee^54^ the same dataset was utilized for training the models. As deep learning models require large datasets for accurate results, but due to the limitation of collecting real data, especially in the medical field, the datasets are synthetically populated in order to provide more data in the training phase of a deep model. However, some studies^23,25,26,29,30,54,55^ have attempted data augmentation with better performance, but our proposed model outperformed the existing ones, as shown in Table 3.

## Conclusion

In this study, a deep learning approach was used to detect BT from the brain images dataset, proposing different CNN based deep learning models such as CNN from scratch, VGG-16, VGG-19, and LeNet-5. During implementation, hyperparameter tuning was performed to choose the best-fit parameters to develop accurate models for detecting BT. Moreover, data augmentation was conducted to add to the number of images synthetically in the existing dataset. Among the proposed models, VGG models outperformed the others with a higher accuracy rate of 99.24% each. The analysis of the reported results demonstrates that the proposed model architectures can learning high classifiable features to distinguish between images containing brain tumor or no tumor compared to existing state-of-the-art CNN based deep learning models. Comparative analysis shows the best fit of the proposed models to be used as a significant CAD system for automatically detecting brain tumors, and can function as a significant decision support system to provide precise decision-making capacity to clinicians diagnosing patients with BT.

It would be better to use heavy database of images that require by the advanced deep learning model such as transformers and Robert model, which could be a potential limitation of this study. However, the study used transfer learning techniques, which are beneficial and achieve optimal results on such size of data. In the future, the dataset could be enhanced by adding real patient images to be acquired from different healthcare and research centers. Several other deep models can be tested with large datasets, and the results can be analyzed for further improvement in automatic detection and diagnosing purposes.
